# Supplementary material for: The angiosperm radiation played a dual role in the diversification of insects and insect pollinators
Source: Nat Commun. 2024 Jan 22;15:552. doi: 10.1038/s41467-024-44784-4 (PMC10803743; doi:10.1038/s41467-024-44784-4)

Supplementary Information

Title

The angiosperm radiation played a dual role in the diversification of insects and insect pollinators

Authors

David Peris[1*] and Fabien L. Condamine[2]

Affiliations

[1]Institut Botànic de Barcelona (CSIC-Ajuntament de Barcelona), 08038 Barcelona, Spain. [2]CNRS, Institut des Sciences de l'Evolution de Montpellier, Université de Montpellier, Place Eugène Bataillon, 34095 Montpellier, France.

* *Corresponding author:* David Peris (david.peris@ibb.csic.es)

The authors contributed equally.

Supplementary Figure 1. Bayesian fossil-based inferences of insect origination (**A**) and extinction (**B**) rates at the family level under the birth-death model with geological epochs as constrained shifts. **C**. The net diversification rates are obtained with the difference between origination and extinction rates (rates below 0 indicate declining diversity). Solid lines indicate mean posterior rates, and the shaded areas show 95% credibility intervals.

A) Origination rate in insects

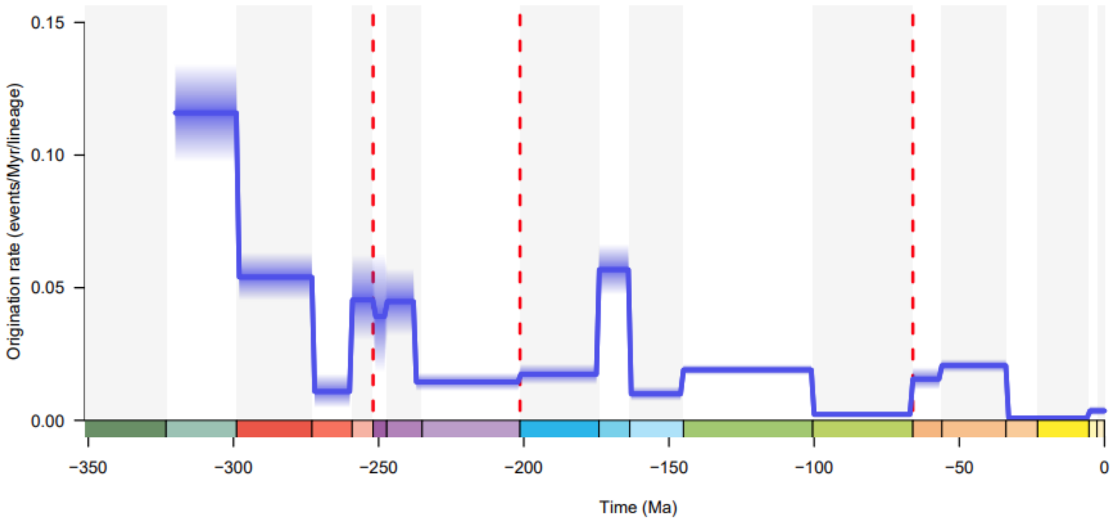

B) Extinction rate in insects

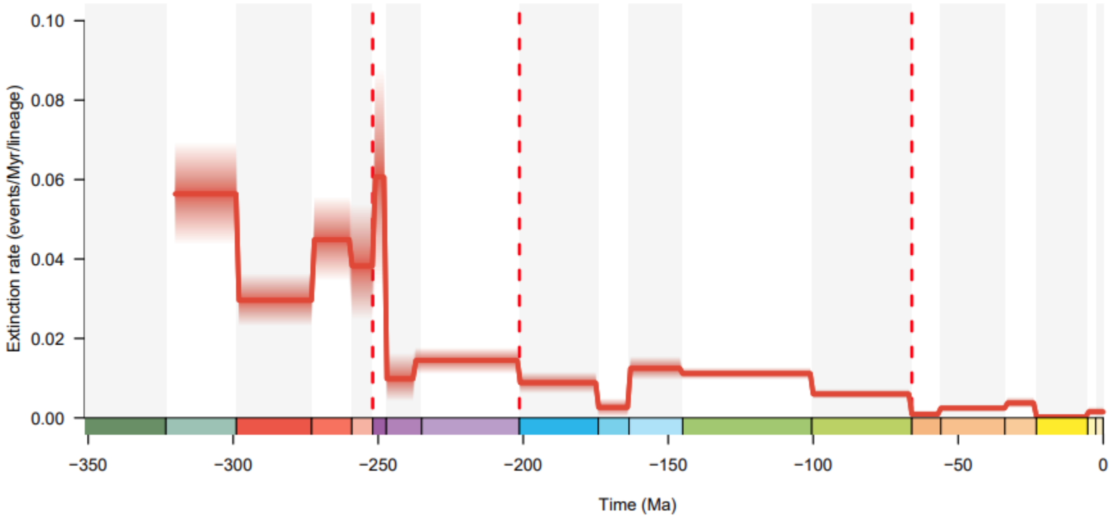

C) Net diversification rate in insects

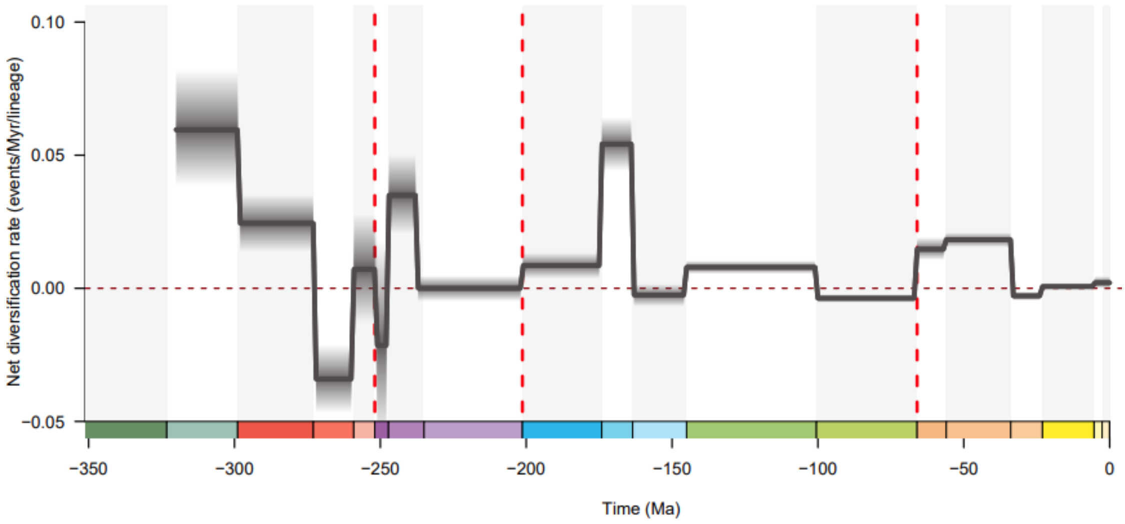

Supplement: Supplementary file 1 — Supplementary Information [file 41467_2024_44784_MOESM1_ESM.pdf]
