# Supplementary material for: The angiosperm radiation played a dual role in the diversification of insects and insect pollinators
Source: Nat Commun. 2024 Jan 22;15:552. doi: 10.1038/s41467-024-44784-4 (PMC10803743; doi:10.1038/s41467-024-44784-4)
Supplement: Supplementary file 3 — Description of Additional Supplementary Files [file 41467_2024_44784_MOESM3_ESM.pdf]

Supplementary Data 1. Results of the multivariate birth-death model applied to all insect families in three set of analyses. The table reports the mean and median posterior parameter estimates (and 95% credibility interval, CI) for the parameters of the MBD model: baseline speciation (λ0), extinction rates (μ0) and correlation parameters (*G*λ and *G*μ) for each of the environmental drivers. Shrinkage weights (ω) greater than 0.5 (highlighted in bold) indicating significant evidence for correlation (positive or negative depending on the respective *G*λ or *G*μ value).

Supplementary Data 2. Results of the multivariate birth-death model applied to pollinator insect families in three set of analyses. The table reports the mean and median posterior parameter estimates (and 95% credibility interval, CI) for the parameters of the MBD model: baseline speciation (λ0), extinction rates (μ0) and correlation parameters (*G*λ and *G*μ) for each of the environmental drivers. Shrinkage weights (ω) greater than 0.5 (highlighted in bold) indicating significant evidence for correlation (positive or negative depending on the respective *G*λ or *G*μ value). Although all environmental variables were significantly correlated with extinction rates ($\omega > 0.5$), the *G*μ correlation parameters had 95% CIs overlapping zero, suggesting a significant effect with a small effect.

Supplementary Data 3. Results of the multivariate birth-death model applied to five selected insect orders in two set of analyses. The table reports the mean and median posterior parameter estimates (and 95% credibility interval, CI) for the parameters of the MBD model: baseline speciation (λ0), extinction rates (μ0) and correlation parameters (*G*λ and *G*μ) for each of the environmental drivers. Shrinkage weights (ω) greater than 0.5 (highlighted in bold) indicating significant evidence for correlation (positive or negative depending on the respective *G*λ or *G*μ value). Although all environmental variables were significantly correlated with extinction rates ($\omega > 0.5$), the *G*μ correlation parameters had 95% CIs overlapping zero, suggesting a significant effect with a small effect.
