# Supplementary material for: The angiosperm radiation played a dual role in the diversification of insects and insect pollinators
Source: Nat Commun. 2024 Jan 22;15:552. doi: 10.1038/s41467-024-44784-4 (PMC10803743; doi:10.1038/s41467-024-44784-4)
Supplement: Supplementary file 5 — Reporting Summary [file 41467_2024_44784_MOESM5_ESM.pdf]

nature portfolio

Corresponding author(s): David Peris

Last updated by author(s): Dec 20, 2023

Reporting Summary

Nature Portfolio wishes to improve the reproducibility of the work that we publish. This form provides structure for consistency and transparency in reporting. For further information on Nature Portfolio policies, see our Editorial Policies and the Editorial Policy Checklist.

Statistics

For all statistical analyses, confirm that the following items are present in the figure legend, table legend, main text, or Methods section.

n/a	Confirmed	
☒	☐	The exact sample size (*n*) for each experimental group/condition, given as a discrete number and unit of measurement
☒	☐	A statement on whether measurements were taken from distinct samples or whether the same sample was measured repeatedly
☒	☐	The statistical test(s) used AND whether they are one- or two-sided<br>*Only common tests should be described solely by name; describe more complex techniques in the Methods section.*
☐	☒	A description of all covariates tested
☒	☐	A description of any assumptions or corrections, such as tests of normality and adjustment for multiple comparisons
☐	☒	A full description of the statistical parameters including central tendency (e.g. means) or other basic estimates (e.g. regression coefficient) AND variation (e.g. standard deviation) or associated estimates of uncertainty (e.g. confidence intervals)
☒	☐	For null hypothesis testing, the test statistic (e.g. *F*, *t*, *r*) with confidence intervals, effect sizes, degrees of freedom and *P* value noted<br>*Give P values as exact values whenever suitable.*
☐	☒	For Bayesian analysis, information on the choice of priors and Markov chain Monte Carlo settings
☒	☐	For hierarchical and complex designs, identification of the appropriate level for tests and full reporting of outcomes
☒	☐	Estimates of effect sizes (e.g. Cohen's *d*, Pearson's *r*), indicating how they were calculated

Our web collection on statistics for biologists contains articles on many of the points above.

Software and code

Policy information about availability of computer code

Data collection	Fossil occurrences of insects were originally compiled in the study of Condamine et al. (2016, in Scientific Reports) using https://paleobiodb.org/ (PBDB). No software or code were used to handle the data collection. The fossil dataset was manually cleaned of synonyms, outdated combinations, nomina dubia, and other erroneous and doubtful records, based on revisions provided in the literature and/or on the expertise of the authors in Condamine et al. (2016). After correction including data addition from the literature, our dataset was composed of 1,527 families of which 671 extant and 856 extinct families, resulting from an in-depth study and curation of the entire bibliography of fossil insects. We then assign each family to know whether it can have been pollinator or not based on the review of Peña-Kairath et al. (2023, in Trends in Ecology and Evolution).
Data analysis	We used PyRate v.3 (https://github.com/dsilvestro/PyRate) to perform all analyses. PyRate is a Bayesian model to estimate origination, extinction and preservation rates from fossil occurrence data.

For manuscripts utilizing custom algorithms or software that are central to the research but not yet described in published literature, software must be made available to editors and reviewers. We strongly encourage code deposition in a community repository (e.g. GitHub). See the Nature Portfolio guidelines for submitting code & software for further information.

Data

Policy information about availability of data

All manuscripts must include a data availability statement. This statement should provide the following information, where applicable:

- Accession codes, unique identifiers, or web links for publicly available datasets
- A description of any restrictions on data availability
- For clinical datasets or third party data, please ensure that the statement adheres to our policy

Datasets (times of origination and extinction of each insect family) are deposited in FigShare.

Human research participants

Policy information about studies involving human research participants and Sex and Gender in Research.

Reporting on sex and gender	N/A
Population characteristics	N/A
Recruitment	N/A
Ethics oversight	N/A

Note that full information on the approval of the study protocol must also be provided in the manuscript.

Field-specific reporting

Please select the one below that is the best fit for your research. If you are not sure, read the appropriate sections before making your selection.

☐ Life sciences ☐ Behavioural & social sciences ☒ Ecological, evolutionary & environmental sciences

For a reference copy of the document with all sections, see nature.com/documents/nr-reporting-summary-flat.pdf

Ecological, evolutionary & environmental sciences study design

All studies must disclose on these points even when the disclosure is negative.

Study description	The key question that we addressed was the role of angiosperms on the insect diversification, for which we hypothesized a positive role on origination rates. We analysed the temporal dynamics – modelling the origination and the extinction – of insects. We investigated which biotic (including clades' interactions) and abiotic factors best explained these processes using a Bayesian fossil-based approach explicitly modelling preservation rates as well as times of origination and extinction of each family.
Research sample	All family-level fossil occurrences for insects known from their oldest record to the present, following the dataset in Condamine et al. (2016, in Scientific Reports).
Sampling strategy	Sample size was determined by the availability of fossil data for insect families. The sample size used in our study corresponds to most of published fossil-based diversification studies. Moreover, the extent of origination and extinction rates and their heterogeneity across insects as inferred using PyRate are sufficiently elevated to allow testing the drivers of diversification and/or extinction.
Data collection	Fossil occurrences of insects were originally compiled in the study of Condamine et al. (2016) using https://paleobiodb.org/ (PBDB). In Condamine et al. (2016), the dataset was cleaned of synonyms, outdated combinations, nomina dubia, and other erroneous and doubtful records, based on revisions provided in the literature and/or on the expertise of the authors. After correction including data addition from the literature, our dataset was composed of 1,527 families of which 671 extant and 856 extinct families, for more than 38,000 occurrences resulting from an in-depth study and curation of the entire bibliography of fossil insects.
Timing and spatial scale	Timing scale of the study spans the origin of insects (330 Ma) to the Present. The spatial scale is global, although the sampling can be considered as biased with the Northern Hemisphere being more sampled (more fossil localities).
Data exclusions	We excluded taxa that are nomina dubia and ichnospecies, which cannot be attributed to a family of insects.
Reproducibility	Our data are not experimental and experiments were thus not replicated. However, we used multiple distinct statistical tests (e.g. environment-dependent diversificaiton models, multivariate birth-death models) and all approaches yielded concordant results.
Randomization	We did not perform an experiment and there was thus no group allocation. We used all insect families for which fossil occurrence data were available. For analyses of clade interactions, there was further group partitioning of data by associating a family to a diet

	when possible (i.e. likely pollinator or not).
Blinding	Blinding was not relevant to our study, because all available data were used (our study did not perform an experiment).

Did the study involve field work? ☐ Yes ☒ No

Reporting for specific materials, systems and methods

We require information from authors about some types of materials, experimental systems and methods used in many studies. Here, indicate whether each material, system or method listed is relevant to your study. If you are not sure if a list item applies to your research, read the appropriate section before selecting a response.

Materials & experimental systems

n/a	Involved in the study
☒	☐ Antibodies
☒	☐ Eukaryotic cell lines
☐	☒ Palaeontology and archaeology
☒	☐ Animals and other organisms
☒	☐ Clinical data
☒	☐ Dual use research of concern

Methods

n/a	Involved in the study
☒	☐ ChIP-seq
☒	☐ Flow cytometry
☒	☐ MRI-based neuroimaging

Palaeontology and Archaeology

Specimen provenance	No new specimens are provided. The fossil datasets are made through a compilation of the fossil data available in Paleobiology Database (https://paleobiodb.org/).
Specimen deposition	All specimens have already been deposited into Museum institutions, and in online database such as Paleobiology Database (https://paleobiodb.org/).
Dating methods	No new dates are provided. Ages of fossil occurrences come from the stratigraphic data bearing the fossils. Chronostratigraphic data come from the International Commission on Stratigraphy and sensu ,Gradstein, F.M, Ogg, J.G., Schmitz, M.D., et al., 2012, The Geologic Time Scale 2012: Boston, USA, Elsevier, https://doi.org/10.1016/B978-0-444-59425-9.00004-4.

☒ Tick this box to confirm that the raw and calibrated dates are available in the paper or in Supplementary Information.

Ethics oversight	NA

Note that full information on the approval of the study protocol must also be provided in the manuscript.
